# Overview of the Genetic Causes of Hereditary Breast and Ovarian Cancer Syndrome in a Large French Patient Cohort

**DOI:** 10.3390/cancers15133420

**Published:** 2023-06-29

**Authors:** Ahmed Bouras, Souhir Guidara, Mélanie Leone, Adrien Buisson, Tanguy Martin-Denavit, Sophie Dussart, Christine Lasset, Sophie Giraud, Marie-Noëlle Bonnet-Dupeyron, Zine-Eddine Kherraf, Damien Sanlaville, Sandra Fert-Ferrer, Marine Lebrun, Valerie Bonadona, Alain Calender, Nadia Boutry-Kryza

**Affiliations:** 1 Laboratory of Constitutional Genetics for Frequent Cancer HCL-CLB, Centre Léon Bérard, 69008 Lyon, France; 2 Team ‘Endocrine Resistance, Methylation and Breast Cancer’ Research Center of Lyon—CRCL, UMR Inserm 1052 CNRS 5286, 69008 Lyon, France; 3 Department of Genetics, Groupement Hospitalier EST, Hospices Civils de Lyon, 69500 Bron, France; souhir.guidara89@gmail.com (S.G.); melanie.leone@lyon.unicancer.fr (M.L.); tanguy.martin-denavit@labo-alpigene.fr (T.M.-D.); sophie.giraud@chu-lyon.fr (S.G.); damien.sanlaville@chu-lyon.fr (D.S.); alain.calender@chu-lyon.fr (A.C.); nadia.boutry-kryza@lyon.unicancer.fr (N.B.-K.); 4 Department of Genetics, CHU Hédi Chaker, Sfax 3027, Tunisia; 5 Department of Biopathology, Centre Léon Bérard, 69008 Lyon, France; adrien.buisson@lyon.unicancer.fr; 6 Center for Medical Genetics, Alpigène, 69007 Lyon, France; 7 Centre Léon Bérard, Unité de Prévention et Epidémiologie Génétique, 69008 Lyon, France; sophie.dussart@lyon.unicancer.fr (S.D.); christine.lasset@lyon.unicancer.fr (C.L.); valerie.bonadona@lyon.unicancer.fr (V.B.); 8 Department of Genetics, Valence Hospital’s Center, 26362 Valence, France; mnbonnet-dupeyron@ch-valence.fr; 9 Institute for Advanced Biosciences, University Grenoble Alpes, INSERM, CNRS, 38000 Grenoble, France; zekherraf@chu-grenoble.fr; 10 UM GI-DPI, University Hospital Grenoble Alpes, 38000 Grenoble, France; 11 Genetics Departement, Centre Hospitalier Métropole Savoie, 73011 Chambery, France; sandra.fert-ferrer@ch-metropole-savoie.fr; 12 Department of Genetics, Saint Etienne University Hospital, 42270 Saint Priez en Jarez, France; marine.lebrun@chu-st-etienne.fr

**Keywords:** HBOC, multigene panel, NGS, RNA analysis, *BRCA* gene

## Abstract

**Simple Summary:**

Hereditary Breast and Ovarian Cancer syndrome (HBOC) is an inherited trait that predisposes adults to an earlier onset of cancer than the general population. HBOC is an autosomal dominant condition caused by heterozygous mutations in one of the HBOC genes. Pathogenic variants in *BRCA1* and *BRCA2* are considered to be the most prevalent causes of HBOC, though mutations on other less common genes have also been described. In 2017, the French Genetic and Cancer Group recommended the screening of 13 genes in individuals with a strong suspicion of HBOC. Here, we report and discuss the results of a retrospective analysis of genetic data related to HBOC gene sequences in a large cohort of 4630 French cases. This work also demonstrated the importance of retesting BRCA1/2 negative cases, as well as the relevance of functional splicing tests in variant classification.

**Abstract:**

The use of multigene panel testing for patients with a predisposition to Hereditary Breast and Ovarian Cancer syndrome (HBOC) is increasing as the identification of mutations is useful for diagnosis and disease management. Here, we conducted a retrospective analysis of BRCA1/2 and non-BRCA gene sequencing in 4630 French HBOC suspected patients. Patients were investigated using a germline cancer panel including the 13 genes defined by The French Genetic and Cancer Group (GGC)—Unicancer. In the patients analyzed, 528 pathogenic and likely pathogenic variants (P/LP) were identified, including *BRCA1* (n = 203, 38%), *BRCA2* (n = 198, 37%), *PALB2* (n = 46, 9%), *RAD51C* (n = 36, 7%), *TP53* (n = 16, 3%), and *RAD51D* (n = 13, 2%). In addition, 35 novel (P/LP) variants, according to our knowledge, were identified, and double mutations in two distinct genes were found in five patients. Interestingly, retesting a subset of BRCA1/2-negative individuals with an expanded panel produced clinically relevant results in 5% of cases. Additionally, combining in silico (splicing impact prediction tools) and in vitro analyses (RT-PCR and Sanger sequencing) highlighted the deleterious impact of four candidate variants on splicing and translation. Our results present an overview of pathogenic variations of HBOC genes in the southeast of France, emphasizing the clinical relevance of cDNA analysis and the importance of retesting BRCA-negative individuals with an expanded panel.

## 1. Introduction

Hereditary Breast and Ovarian Cancer syndrome (HBOC) is an autosomal dominant inherited cancer predisposition characterized by an increased risk of breast and ovarian cancers. It represents about 10–15% and 25% of all breast and ovarian cancers, respectively [1]. The *BRCA1* (OMIM # 113705) and *BRCA2* (OMIM# 612555) genes are the most common and exhaustively studied, with a lifetime risk of developing cancer for BRCA mutation carriers of 60–80% for breast and 20–40% for ovarian cancers [2]. 

The recent development and availability of high-throughput sequencing (HTS) technologies have revolutionized the molecular diagnosis of inherited cancers. Today, many clinical laboratories routinely perform multigene panel testing for the molecular diagnosis of HBOC [3,4]. The French Genetic and Cancer Group (GGC)—Unicancer—an expert group that creates guidelines for the detection and the prevention of hereditary cancers in affected families, selected a set of 13 genes to be included in an HBOC diagnosis panel: *BRCA1*, *BRCA2*, *PALB2*, *TP53*, *CDH1*, *PTEN*, *RAD51C*, *RAD51D*, *MLH1*, *MSH2*, *MSH6*, *PMS2*, and *EPCAM* [5]. Among these, 5–10% of familial breast cancers are, for instance, attributed to mutations in genes such as *TP53* and *PTEN* [6]. Moreover, a recent meta-analysis provided evidence supporting the pathogenicity of *BRIP1*, *RAD51C*, and *RAD51D* mutations in relation to ovarian cancer, cumulatively contributing to ~2% of ovarian cancer cases [7]. 

Here, we retrospectively analyzed 13 genes involved in HBOC with an (NGS)-based multigene panel, including flanking and coding regions of *BRCA1*, *BRCA2,* and 11 other genes from the panel recommended by the GGC. The purpose of our study was to identify the mutational spectrum of HBOC in a cohort of 4630 French probands with an indication for HBOC gene analysis and to determine the value of retesting BRCA1/2 negative cases using an expanded panel of breast and ovarian cancer genes. We also analyzed exon splicing patterns in four potentially spliceogenic variants to assess their deleterious effects. The obtained results are extensively discussed, with remarks on the novel findings.

## 2. Materials and Methods

### 2.1. Patients

A total of 4630 patients were recruited from several Oncogenetic Departments with a predisposition for HBOC in France between 2017 and 2020, according to national recommendations. Only patients with an Eisinger score ≥ 4 or those eligible for anti-PARP therapy were considered in this study [8]. Two samples were collected from each subject: peripheral blood in EDTA and a buccal swab transferred to FTA paper. Total genomic DNA was extracted using the automated procedure implemented on the STARlet platform (Hamilton Company, Reno, NV, USA). Written informed consent was obtained from all subjects.

### 2.2. NGS Analysis

The 4630 patients were analyzed using the Hereditary Cancer Solution (HCS) CE-IVD kit (SOPHiA GENETICS, Saint-Sulpice, Switzerland) according to the manufacturer’s recommendations. Briefly, 200 ng of gDNA was enzymatically digested and underwent end repair and A-tailing. Libraries were quantified using a Qubit dsDNA HS Assay Kit on a Qubit 2.0 fluorometer (Thermo Fisher Scientific, Invitrogen, Villebon sur Yvette, France). The library size was verified using capillary electrophoresis (2200 TapeStation, Agilent Technologies, Santa Clara, CA, USA). Sequencing was performed on a 600-cycle format V3 flow cell with an Illumina MiSeq DX (Illumina, San Diego, CA, USA). Each run included 47 patients and a positive control. SOPHiA DDM platform based on SOPHiA artificial intelligence (AI) was used for processing sequencing data. Sophia DDM can detect single nucleotide variants (SNVs), indels, copy number variations (CNVs), and Alu insertions. Genes analyzed in this study are represented in Table 1.

### 2.3. Variant Interpretation 

Human Genome Variation Society (HGVS) guidelines were used for variant nomenclature. Variant numbering was based on the coding sequence. Therefore, the first nucleotide position was attributed to the A of the ATG translation initiation codon.

Pathogenic and likely pathogenic variants (P/LP) were classified with a multifactorial model including cosegregation data (French national COVAR study) [9]. Variant interpretation was guided by pathogenicity-predicted scores using several in silico prediction tools: (CADD) algorithm [10], AlignGVGD [11], SIFT [12], and MutationTaster2 [13]; SPIP [14]; and Splice AI [15].

### 2.4. Additional Analyzes

All pathogenic and likely pathogenic variants were confirmed on a second sample by a second appropriate technique. Sanger sequencing was performed on ABIPrism 3130XL/3730 Genetic Analyzers (Thermo Fisher Scientific) and was used for SNVs and Indel confirmation. CNVs were confirmed by Multiplex Ligation-Dependent Probe Amplification (MLPA) Analysis (MRC Holland, Amsterdam, The Netherlands). Complex variants such as Alu insertion were confirmed by PCR with specific conditions followed by Sanger sequencing. 

Variants potentially affecting splicing were studied by RT-PCR. Briefly, RNA was extracted from PaxGene samples or EBV-immortalized lymphoblastoid cells, reverse transcribed into cDNA (Superscript III First-Strand Synthesis SuperMix, Invitrogen, Villebon sur Yvette, France), and amplified for the region of interest. All amplifications were performed in triplicate. cDNA PCR primers are presented in the Supplementary Table S1. Interpretation of results was carried out according to the recommendations of the French GGC Unicancer [16].

## 3. Results

The clinical characteristics of the patients analyzed (when available) are shown in Table 2.

### 3.1. Multigene Panel Screening

**Figure 1:**
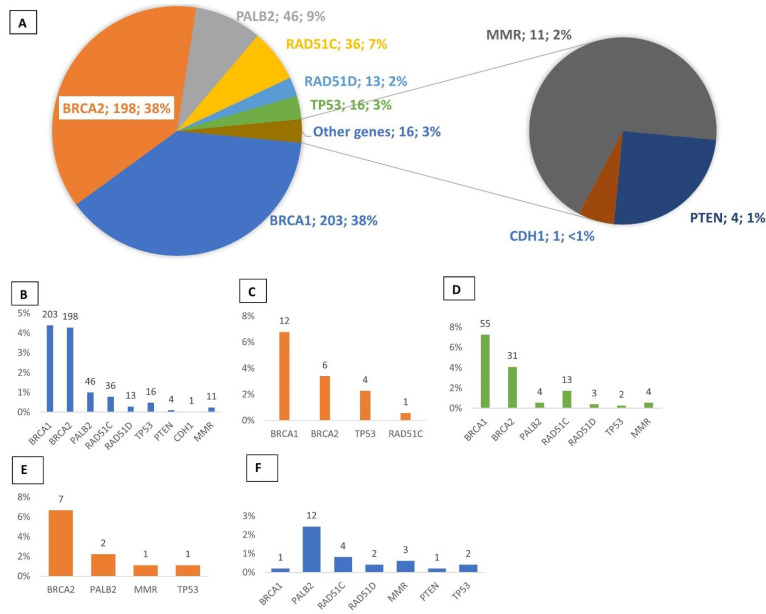
Distribution of pathogenic or likely pathogenic (P/LP) variants detected by NGS. (**A**) Distribution and percentages of the 530 (P/LP) variants detected in all HBOC cases. (**B**) Frequencies of (P/LP) variants in all HBOC cases. (**C**) Genes with (P/LP) variants detected in the 177 patients with breast cancer diagnosed before 31 years of age. (**D**) Genes with (P/LP) variants detected in the 759 patients with ovarian cancer. (**E**) Genes with (P/LP) variants detected in 91 patients with male breast cancer (**F**). Genes with (P/LP) variants detected in the 492 patients who had previously tested negative for BRCA pathogenic variants and retested using the GGC-HBOC gene panel.

From the 4630 patients analyzed by NGS, 528 P/LP variants (pathogenic or likely pathogenic, classes 5 or 4) were identified in HBOC genes, including *BRCA1* (n = 203, 38%), *BRCA2* (n = 198, 37%), *PALB2* (n = 46, 9%), *RAD51C* (n = 36, 7%), *TP53* (n = 16, 3%), and *RAD51D* (n = 13, 2%). Less than 3% of the remaining mutations were found in other HBOC predisposing genes: *PTEN, CDH1,* and mismatch repair genes (*MLH1, MSH2, MSH6, PMS2*) (Figure 1A,B). From the 177 patients diagnosed with breast cancer, 23 (P/LP) variants were detected in young individuals under 31 years of age. BRCA1/2 mutations were found in 18 patients (10.2%), followed by *TP53* (n = 4, 2.3%) and *RAD51C* (n = 1, 0.6%) (Figure 1C). In ovarian cancer patients, 112 (P/LP) variants were identified. BRCA1/2 mutations were detected in 11.3% of patients (n = 86), and sixteen RAD51C/D mutations were identified (2.1%). Mutations in other genes were found in 1.3% of patients, including *PALB2* (n = 4), MMR (n = 4), and TP53 (n = 2) (Figure 1D). Among the 91 men diagnosed with breast cancer, 12 (P/LP) variants were identified (13%); 7/91 displayed a mutation in BRCA2 and 2/91 in PALB2 (Figure 1E). Among the 492 patients who had previously tested negative for BRCA pathogenic variants and retested using the GGC-HBOC gene panel, 25 (P/LP) variants were identified (5%), of which nearly half were associated with *PALB2* (n = 12/25). Additionally, NGS analysis revealed a pathogenic *BRCA1* variant: c.2231_3354del: p. (Ala744Aspfs*3) in a patient previously classified as BRCA-negative by Sanger sequencing and MLPA analysis (Figure 1F).

### 3.2. Novel Mutations

Thirty-three novel pathogenic variants (class 5 or 4) were identified in patients of our cohort. Eight variants were identified in *BRCA1*, 11 in *BRCA2*, 11 in *PALB2,* and 5 in RAD51C/D (Figure S1).

### 3.3. Recurrent Mutations

Recurrent mutations are represented in Table 3.

*BRCA1* families (n = 203) carried a total of 126 different mutations. Three recurrent mutations (c.5266dup, c.3481_3491del, and c.1115G>A) were observed, representing 27% of *BRCA1* mutations. Among *BRCA2*-positives families (n = 198), 118 different mutations were observed. Variant c.4889C>G was highly represented (11 unrelated families). The five most frequent pathogenic variants accounted for approximately 30% of *BRCA2* mutations. The two most recurrent pathogenic mutations of *RAD51C* were located in splice sequences (c.1026+5_1026+7delGTA and c.965+5G>A) and represented 44% of all *RAD51C* mutations (n = 36). The two recurrent mutations observed in *RAD51D* were c.803G>A and c.170del. They represented 70% of all *RAD51D* mutations (n = 13). For *PALB2,* two recurrent mutations were observed (c.2257C>T and c.1915G>T), each representing 7% of *PALB2* mutations (n = 46).

### 3.4. Identification of Double Mutations

A double mutation in two distinct genes was detected in five patients. These double mutations include the following combinations: *BRCA1*/*BRCA2*, *BRCA1*/*RAD51C*, *BRCA2*/*RAD51C,* and *BRCA2*/*PTEN*. The carrier of the *BRCA2*/*PTEN* mutation presented an epithelial thyroid cancer and a breast cancer (Table 4).

### 3.5. mRNA Transcript Analysis of Patients with P/LP Splice Variants

To detect variants that may affect splicing, we used in silico prediction tools such as Alamut Visual v2.15 (Interactive Biosoftware), SPIP predictions [14], and Splice AI prediction [15]. Overall, four variants located in intronic splicing regions were identified and suspected of being highly deleterious: *PALB2* c.1631_1684+1846del, *RAD51C* c.145+3A>C, *RAD51C* c.905-2del, and *PALB2*: c.3350+4A>G.

**Figure 2:**
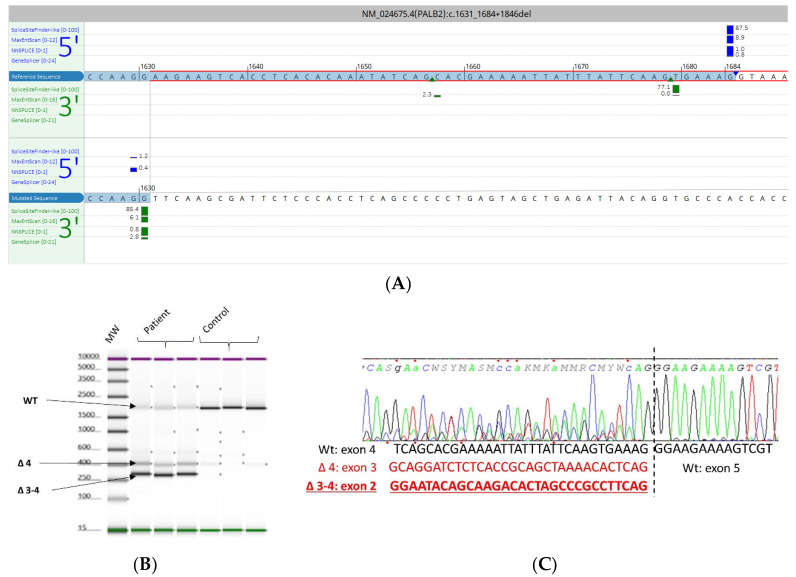
The PALB2 c.1631_1684+1846del variant identified in the proband of the HBOC family. (**A**) In silico splicing analysis using Alamut Visual plus v1.7.1 (Interactive Biosoftware). (**B**) RT-PCR from lymphocyte-derived RNA. Automated gel electrophoresis using the TapeStation detection system from patient and control samples. Two additional bands were observed in patient samples which were absent in the negative control. (**C**) Electropherogram related to Sanger sequencing (reverse sequence) of these amplicons demonstrates the abnormal structure of the two corresponding transcripts reflecting by exon 4 or exons 3–4 skipping during splicing. The sequence of the wild-type transcript is represented in black while sequences of aberrant transcripts are represented in red.

The *PALB2* c.1631_1684+1846del variant consisted of a deletion of a sequence over 1.8 Kbp in size, encompassing the consensus splice donor site of exon 4. To evaluate the splicing impact of this variant, we performed RT-PCR from lymphocyte-derived mRNA. Primers were designed in exons 2 and 5 of *PALB2*. We observed, in addition to the expected band, two smaller bands in patient samples. Sanger sequencing of the PCR products showed that these additional fragments corresponded to abnormal transcripts with skipping of exon 4 and exons 3–4 (Figure 2).

**Figure 3:**
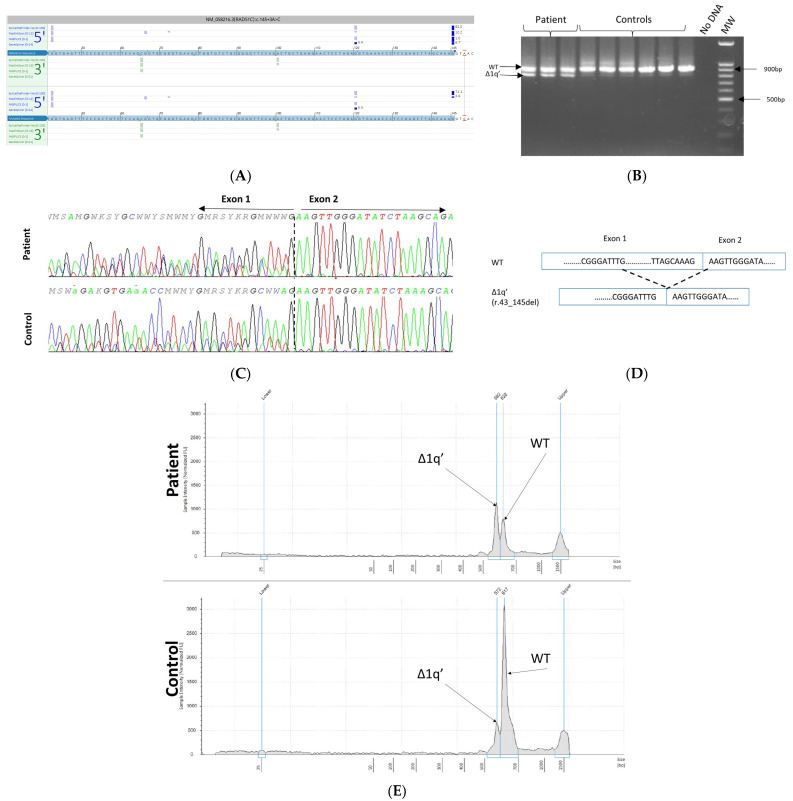
The RAD51C c.145+3A>C variant identified in the proband of HBOC family. (**A**) In silico splicing analysis using Alamut Visual Plus v1.7.1 (Interactive Biosoftware). (**B**) RT-PCR of lymphocyte-derived RNA gel electrophoresis from patient and control samples in triplicate. Electrophoresis of RT-PCR products demonstrates an additional band (820 bp), as well as the expected band (923 bp), in the proband. The density of the aberrant band is much stronger in the proband than in the controls. (**C**) Sequencing of the RT-PCR products confirmed the significant increase in the aberrant transcript Δ1q’ (r.43_145del). (**D**) Schematic representation of the two transcripts observed. (**E**) Tapestation analysis of the two transcripts observed in the patient and a normal control sample.

The RAD51C c.145+3A>C variant was detected in a proband diagnosed with ovarian cancer at 63 years. This variant is absent from the gnomAD population database (v2.1.1) and has never been reported in the literature. Our tools predicted a loss or a weakening effect on the canonical donor site in intron 1. EBV-immortalized lymphoblastoid cells were used for RNA analysis. PCR was designed to generate a fragment that spanned part of the 5′UTR and exon 6. The primers used are listed in Supplementary Table S1. RT-PCR analysis and Sanger sequencing revealed that the intronic variant resulted in a marked increase in an alternative transcript r.43_145del missing the 3′ end of the first exon. As shown in Figure 3, the c.145+3A>C variant activates a cryptic 5′ splice donor site at the c.43G position resulting in the deletion of 103 bp and presumably leading to a frame-shift p.Val15Lysfs*10. The alternative transcript Δ1q’(r.43_145del) is present physiologically at a rate of 10% [17].

**Figure 4:**
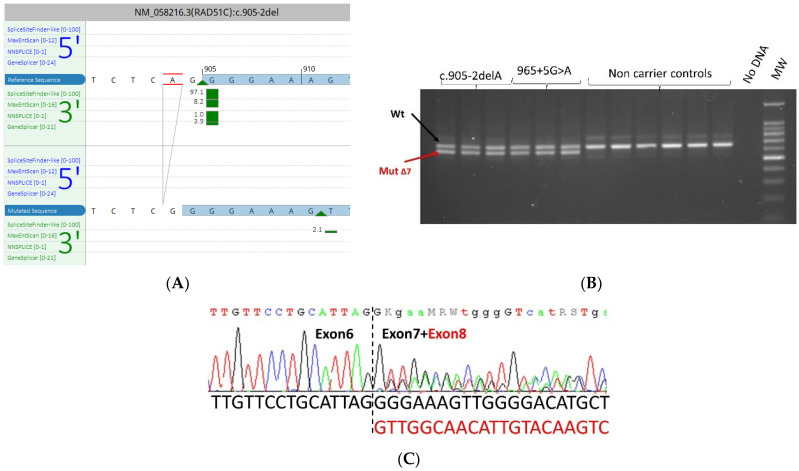
The RAD51C: c.905-2delA variant identified in the proband of HBOC family. (**A**) In silico splicing analysis using Alamut Visual Plus v1.7.1 (Interactive Biosoftware). (**B**) RT-PCR of lymphocyte-derived RNA. Electrophoresis of RT-PCR products demonstrates an additional band (604 bp), as well as the expected wild-type band (664 bp), in the proband and in the positive control (carrier of the c.965+5G>A). The density of the aberrant band is comparable to the wild-type band. (**C**) Electropherogram showing that the variant causes an aberrant transcript corresponding to exon 7 skipping in the patient sample (forward). The sequence of the wild-type transcript is represented in black while the sequence of the aberrant transcript is represented in red.

*RAD51C* c.905-2del was detected in co-occurrence with a pathogenic *BRCA2* mutation c.9097dupA p.(Thr3033fs) in a woman who developed breast cancer at 55. In silico tools predicted the total abolition of the splice acceptor site. To assess the splicing effects of this variant, mRNA was studied by RT-PCR targeting exons 4 to 9 in the carrier of the variant. We used as a positive control a patient carrying the RAD51C 965+5G>A variant known to cause skipping of exon 7 [18]. The results showed, in addition to the expected band, a smaller band in the patient and the positive control corresponding to the transcript with exon 7 skipping (Figure 4).

**Figure 5:**
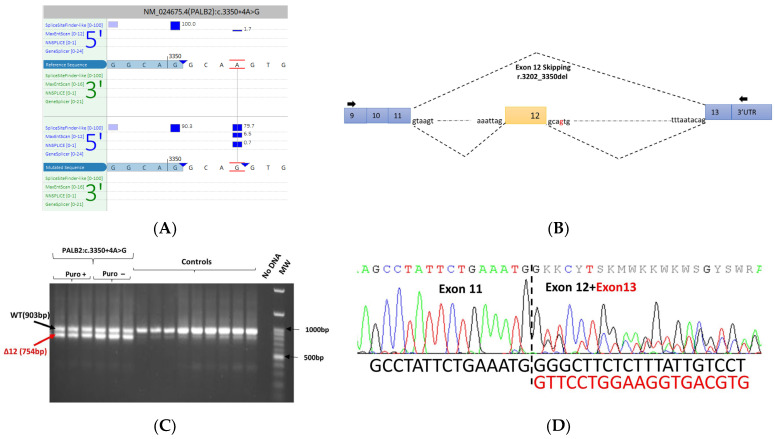
The PALB2 c.3350+4A>G variant identified in the proband of HBOC family. (**A**) In silico splicing analysis using Alamut Visual Plus v1.7.1 (Interactive Biosoftware). (**B**) Schematic representation of the aberrant transcripts observed. (**C**) RT-PCR of lymphocyte-derived RNA in the absence and in the presence of an NMD inhibitor (puromycin). Electrophoresis of RT-PCR products demonstrates an additional band (754 bp), as well as the expected wild-type band (903 bp), in the proband. The density of the aberrant band is comparable to the wild-type band. (**D**) Electropherogram showing that the variant causes an aberrant transcript corresponding to the transcript with exon 12 skipping in the patient sample (forward). The sequence of the wild-type transcript is represented in black while the sequence of the aberrant transcript is represented in red.

The *PALB2* c.3350+4A>G variant was detected in a proband diagnosed with an invasive lobular carcinoma at 49. The sister of the proband developed an invasive lobular carcinoma at 42. This variant is predicted to create a de novo splice site and has been previously reported in the literature to induce splicing defects when tested using a minigene assay [19]. To confirm the splicing effects of this variant in this independent study, mRNA was analyzed by RT-PCR targeting exon 9-3′UTR. In addition to the expected wild-type band, an additional smaller band was observed in the patient sample and absent in the control sample. Sanger sequencing of PCR products showed that the additional band observed in the patient sample corresponded to an abnormal transcript lacking the exon 12 (Figure 5).

## 4. Discussion

The identification of pathogenic variants in high-risk individuals of developing breast and ovarian cancers is improving cancer detection and prevention in HBOC families. The recent development and availability of high throughput sequencing (HTS) techniques, such as multigene panel testing (MPT), has led to the discovery of novel monogenic causes of cancer. The implementation of such an MPT-based strategy in clinical practice enhances the genetic diagnosis of cancer and optimizes the management and care of patients affected or predisposed to developing hereditary cancer [20]. In this work, we investigated a French cohort of 4630 high-risk HBOC patients using an MPT-based strategy. Overall, the diagnostic yield of genetic testing, which is based on the identification of pathogenic and likely pathogenic variants, was 11.5%. This result is comparable with those reported in the literature by other independent studies [3,21]. Among the 13 genes tested in our panel, and as expected, mutations in *BRCA1* and *BRCA2* were predominant and accounted for 75% of all mutations detected in our cohort. In addition to *BRAC1*/2 variants, we identified deleterious variants in other canonical HBOC genes like *PALB2* (n = 46; 9%), *RAD51C* (n = 34; 7%), and *TP53* (n = 16; 3%). Their collective contribution was estimated at around 2% of all patients analyzed in this study and provided further evidence of the heterogeneous genetic component underlying HBOC [22].

It is well known that breast cancer can be associated with several other syndromes like Cowden syndrome. The four patients harboring pathogenic *PTEN* variants displayed personal and familial clinical presentations similar to that of Cowden (Supplementary Table S2) syndrome. The inclusion of *PTEN* in the HBOC gene panel is useful in the case of missed diagnoses of Cowden syndrome, which is likely underdiagnosed due to the high phenotypic heterogeneity, and the high frequency in the general population of certain clinical manifestations [23].

Women who harbor *TP53* mutations were reported to have an increased risk of developing early-onset breast cancer [24]. In our cohort of patients with breast cancer ≤ 31 years, P/LP variants were found in 13% of cases, the majority of which were identified in *BRCA1*, *BRCA2,* and *TP53* (6.8%, 3.4%, and 2.3%, respectively). Our diagnostic yield of TP53 testing in early-onset breast cancer patients is comparable with a study conducted by Bakhuizen et al. in a large national cohort of 370 women diagnosed with breast cancer before the age of 31 [25] and the result of a British multicenter study with an overall detection rate of the TP53 PV/LPV germline variant of 3.3% in all women diagnosed with breast cancer at <30 years of age [26]. In patients with germline TP53 mutations, several studies have shown the risk of secondary tumors after radiotherapy, suggesting that radiotherapy should be avoided in a breast cancer patient with a germline TP mutation [27,28,29]. Interestingly, most TP53 mutations were found in patients not meeting the Li-Fraumeni criteria, highlighting the broad clinical spectrum associated with TP53 mutations.

For the 759 patients with a personal and/or familial history of only ovarian cancer, as expected, BRCA1/2 genes were the most frequently mutated, followed by *RAD51C* and *RAD51D*. The association between RAD51C/D pathogenic mutations and ovarian cancer has already been established in several studies [30]. The increased risk of ovarian cancer in carriers of MMR mutation justifies their inclusion into our MPT. In our series, we detected four deleterious MMR mutations in families (0.5%) without Amsterdam or Bethesda Guidelines (Supplementary Table S3). This low rate of MMR mutation detection is offset by the medical benefit resulting from their identification [31]. Indeed, the identification of patients carrying an MMR mutation with ovarian cancer could improve their management by making them eligible for immunotherapy [32].

In our study, 12% of male patients with breast cancer (MBC) were mutated. As expected, *BRCA2* was the most mutated gene in the cohort, followed by *PALB2*. Our results are consistent with other studies that included MBC [33,34]. According to the 2021 National Comprehensive Cancer Network (NCCN) Genetic/Familial High-Risk Assessment guidelines, men with a germline BRCA (P/LP) variant are advised to begin breast self-examination and annual clinical breast exam at the age of 35 and to consider annual mammogram screening if they have gynecomastia, from the age of 50 or ten years before the earliest known case of male breast cancer in the family [32]. However, there are no recommendations for males who carry germline (P/LP) variants in non-BRCA genes. A study by Chamseddine et al. highlighted that *BRCA2* is not the only gene associated with MBC, as other genes like *PALB2* may also be linked to MBC [35].

Among the 492 patients who had negative results by Sanger sequencing and MLPA analysis for the *BRCA1* and *BRCA2* genes, P/LP variants were identified in 5% of the studied cohort. We found a high frequency (2.5%) of pathogenic mutations in the *PALB2* gene, which is in accordance with previous independent studies conducted by other teams [36,37]. Surprisingly, MPT revealed a pathogenic variant in *BRCA1* in a patient initially classified as BRCA-negative following targeted Sanger sequencing and MLPA analysis. The *BRCA1* variant: c.2231_3354del; p.(Ala744Aspfs*3) is a large intragenic deletion (>1 Kb) not detectable by the Sanger method. Using MLPA (Multiplex Ligation-dependent Probe Amplification), we failed to detect this deletion because it was uncovered by the available probes. Our findings indicate that retesting *BRCA1/2*-negative individuals with an expanded panel of 13 genes could produce clinically relevant results, which is consistent with the study of Jordan Lerner-Ellis et al. in a Canadian Hospital [38].

In the present study, we identified double mutations in five patients. These identifications improve the phenotype-genotype correlation of some double mutations, thus optimizing genetic counseling. A double mutation in *BRCA2* and *PTEN* was found in a patient with a history of epithelial thyroid cancer at the age of 29 treated by thyroidectomy. The indication of MPT was retained after a breast cancer was diagnosed at the age of 37. These two tumors are part of the spectrum of PTEN Hamartoma Tumor Syndrome (PHTS) [39]. The discovery of this double mutation made it possible to find the parental branch carrying the *PTEN* variant and to offer adapted genetic counseling for mutated relatives.

We performed mRNA transcription analysis on four variants predicted to impact splicing by in silico tools. The four variants showed abnormal transcriptional fragments and were classified as pathogenic. The *PALB2* c.1631_1684+1846del variant led to the production of two abnormal transcripts, one of which consists of an in-frame deletion encompassing a large part of the chromatin-association motif (Cham) functional domain [40]. The RAD51C c.905-2del variant was previously studied by an in vitro minigene experiment that produced similar results [41].

The *PALB2* c.3350+4A>G causes the skipping of exon 12, which encodes part of the important WD40 domain [42]. However, discrepancies were found between our result and a minigene RNA test. Valenzuela-Palomo et al. showed that the *PALB2* c.3350+4A>G variant predominantly resulted in an out-of-frame transcript with 4 bp intronic retention in addition to a very small amount of the ∆E12 transcript. This may be due to the complexity of alternative splicing patterns in some genomic regions as described for the BRCA2 variant c.7976+5G>T [43]. These differences in the RNA tests do not impact the final classification of the variant, which has been classified as pathogenic. Indeed, in addition to its effect on splicing, this variant has been reported in the literature in the compound heterozygous state with a frame-shift variant in a child who was affected with Fanconi Anemia of Type N and who developed medulloblastoma in early childhood [44]. Nevertheless, this case underlines the importance of using patient biological material in addition to minigene tests in order to avoid misinterpretation.

This retrospective study showed some limitations, such as a lack of detailed clinical description and/or familial segregation data for some probands, as well as the exclusion of some moderate penetrance HBOC genes which is somewhat controversial (e.g., ATM, CHEK2) [3,45]. In fact, the French GGC does not retain these genes in the diagnostic panel requiring additional knowledge for the moment. These genes are currently included in the national TUMOSPEC research protocol, which was designed to estimate the cumulative cancer risk for carriers of P/LP variants in such genes usually tested in the context of HBOC [46].

## 5. Conclusions

Overall, our study, which includes the description of the mutational spectrum of more than 4500 probands, is one of the most important French cohorts reported in HBOC since the publication of the French-GGC guidelines in 2017 [7]. Our study suggests that an NGS approach based on a multigene panel provided a rapid, inexpensive, and highly efficient workflow for the identification of genomic variants in the most important HBOC genes. We also demonstrated that a multigene retesting approach could result in the identification of clinically relevant variants in BRCA1/2 and non-BRCA1/2 genes at a sufficiently high yield in appropriately selected patients. This work also illustrated the importance of functional RNA analysis to determine VUS classification in hereditary cancer syndromes.

## Figures and Tables

**Table 1:** List of HBOC genes screened in this study.

Gene	Transcript	Gene	Transcript
*BRCA1*	NM_007294.3	*CDH1*	NM_004360.4
*BRCA2*	NM_000059.3	*MLH1*	NM_000249.3
*PALB2*	NM_024675.3	*MSH2*	NM_000251.2
*RAD51C*	NM_058216.2	*MSH6*	NM_000179.2
*RAD51D*	NM_002878.3	PMS2 *	NM_000535.6
*TP53*	NM_000546.5	EPCAM **	NM_002354.2
*PTEN*	NM_000314.6		

* Due to the high sequence similarity shared with the paralog, the detection of variants and CNVs of exons 12 to 15 of *PMS2* was not carried out. ** Only large genomic rearrangements were investigated.

**Table 2:** Clinical data of the patients studied.

Personal and Familial Criteria	Total	Detection Rate of P/LP Variants, n (%)
Breast carcinoma ≤ 31 years	177	23 (13%)
Breast carcinoma ≤ 36 years	564	23 (14%)
Male breast carcinoma	91	11 (12%)
Ovary adenocarcinoma	759	112 (15%)
Bilateral breast carcinoma	244	33 (14%)
Free of cancer index case with a strong familial history of HBOC	108	9 (8.3%)
Index case with both breast and ovarian cancer	74	9 (17.6%)
Patients previously tested negative for BRCA pathogenic variants retested using the panel of 13 HBOC genes	492	25 (5%)

n: number of patients; %: percentage of patients; P/LP: pathogenic/likely pathogenic.

**Table 3:** Recurrent pathogenic variants identified in our study.

Genes	cDNA Position	Protein	Frequency	%
*BRCA1*	c.5266dup	p.(Gln1756Profs*14)	15	7.4
	c.3481_3491del	p.(Glu1161Phefs*3)	10	5
	c.1115G>A	p.(Trp372*)	9	4
	c.4391del	p.(Pro1464Leufs*2)	5	2
	c.4327C>T	p.(Arg1443*)	4	2
	c.3756_3759delGTCT	p.(Ser1253Argfs*10)	4	2
	c.191G>A	p.(Cys64Tyr)	4	2
*BRCA2*	c.4889C>G	p.(Ser1630*)	11	5
	c.3847_3848delGT	p.Val1283Lysfs*2	7	4
	c.9294C>A	p.(Tyr3098*)	6	3
	c.1813dupA	p.(Ile605Asnfs*11)	6	3
	c.1310_1313delAAGA	p.(Lys437Ilefs*22)	6	3
	c.7680dup	p.(Gln2561Serfs*5)	5	3
	c.8364G>A	p.Trp2788*	4	2
	c.2612C>A	p.(Ser871*)	4	2
	c.5909C>A	p.(Ser1970*)	4	2
*RAD51C*	c.1026+5_1026+7delGTA	p.(Arg322Serfs*22)	9	25
	c.965+5G>A	p.(Glu303Trpfs*41)	7	19
*RAD51D*	c.803G>A	p.(Trp268*)	5	40
	c.170del	p.(Leu57Argfs*10)	4	30
*PALB2*	c.2257C>T	p.(Arg753*)	3	7
	c.1915G>T	p.(Glu639*)	3	7

Note: %: percentage of recurrent mutations compared to the total number of mutations observed in the same gene.

**Table 4:** Double heterozygous pathogenic variants identified in our study.

Gene	Diagnosis	Variant	Protein Effect	Class
Patient 1				
*BRCA1*	Uterus cancer at 30 years. Triple-negative breast carcinoma at 33 years	c.5309G>T	p.(Gly1770Val)	5
*BRCA2*		c.7234_7235insG	p.(Thr2412Serfs*2)	5
Patient 2				
*BRCA1*	Breast carcinoma at 36 years, contralateral triple-negative breast carcinoma at 62 years	c.212+3A>G	p.Cys64fs*	5
*RAD51C*		c.1026+5_1026+7delGTA	p.(Arg322Serfs*22)	5
Patient 3				
*BRCA2*	Breast carcinoma at 55 years	c.9097dupA	p.(Thr3033fs)	5
*RAD51C*		c.905-2del	p.(Glu303Trpfs*41)	5
Patient 4				
*BRCA2*	Ovarian cancer	c.1842dupT	p.(Asn615*)	5
*RAD51C*		c.773G>A	p.(Arg258His)	5
Patient 5				
*BRCA2*	Epithelial thyroid cancer at 29 years Breast carcinoma at 37 years	c.3645_3646delGTinsTAAAAAG	p.(Phe1216Lysfs*14)	5
*PTEN*		c.1003C>T	p.(Arg335*)	5

## Data Availability

The data presented in this study are available on request from the corresponding author. The data are not publicly available due to restrictions of patient privacy.

## Supplementary Materials

The following supporting information can be downloaded at: https://www.mdpi.com/article/10.3390/cancers15133420/s1: Figure S1: Lolliplot representing BRCA1, BRCA2, PALB2, RAD51C, and RAD51D proteins with novel variants reported; Table S1: Primer Sequences used for the RNA Analysis; Table S2: Summary of carriers of (likely) pathogenic PTEN variant; Table S3: Summary of ovarian cancer patients’ carriers of (likely) pathogenic in MMR genes.
