# Supplementary material for: Overview of the Genetic Causes of Hereditary Breast and Ovarian Cancer Syndrome in a Large French Patient Cohort
Source: Cancers (Basel). 2023 Jun 29;15(13):3420. doi: 10.3390/cancers15133420 (PMC10341368; doi:10.3390/cancers15133420)
Supplement: Supplementary file 1 [file cancers-15-03420-s001.zip › cancers-2443339-supplementary.pdf]

Supplementary Table S1 : Primer Sequences used for the RNA Analysis

Variants	Forward	Reverse
RAD51C c.145+3A>C	T1F : CTCCGGGGTTAGCAGGTG	T6RAGGAACAAGCAAGGCCTGAT
PALB2 c.1631_1684+1846del	T2F : TACAGCAAGACACTAGCCCG	T5R :TTGACTCAAAGGGCTCCACT
PALB2 :c.3350+4A>G	T9F : GTTAGTAGCAGTGGGACCCT	T13R :TGCCATTTGAAGCTTTATGTACA
RAD51C :c.905-2delA	T4F : CGAAAAGCTTTGGAGGATTTCAC	T9R : TTCATTCATGCCATAGTGTGTTT

Supplementary Table S2: Summary of carriers of (likely) pathogenic *PTEN* variant

**Patient**	**Mutation**	**diagnosis**	**symptoms in relatives**
S1	**c.493-1G>C p.?**	-papillary thyroid carcinoma (23)<br>-meningioma (40)<br>-bilateral breast cancer (56)	-mother: Breast cancer (50)<br>-sister: breast cancer (44)
S2	**c.389G>C p.(Arg130Pro)**	-Breast cancer (48)<br>- macrocephalia<br>- thyroid nodules	-father: polyposis<br>-paternal aunt: thyroid nodules + breast cancer
S3	**c.389G>C p.(Arg130Pro)**	-polyposis (33)<br>-endometrial cancer (34)<br>-breast cancer (46)	-mother: polyposis+ leiomyoma + thyroid nodules + breast cancer (54)<br>-sister: bilateral breast cancer (34)
S4	**c.1003C>T p.(Arg335*)**	- epithelial thyroid cancer (29)<br>-breast carcinoma (37)	-mother: bilateral breast cancer (37+41)

Supplementary Table S3: Summary of ovarian cancer patients' carriers of (likely) pathogenic in MMR genes

**Variant**	**Proband**	**relatives**
MSH6 : Exon1-Exon4 deletion	OV (58)	Father : Bladder Cancer (90)<br>Mother : Colorectal cancer (84)
PMS2: c.137G>T p.(Ser46Ile)	OV (50)	Sister : skin lymphosarcoma (50)
MSH6 : c.123_124del p.(Pro42Phefs*47)	OV (62)	/
MLH1 : c.677+3A>G	OV (?)	/

OV : ovarian cancer

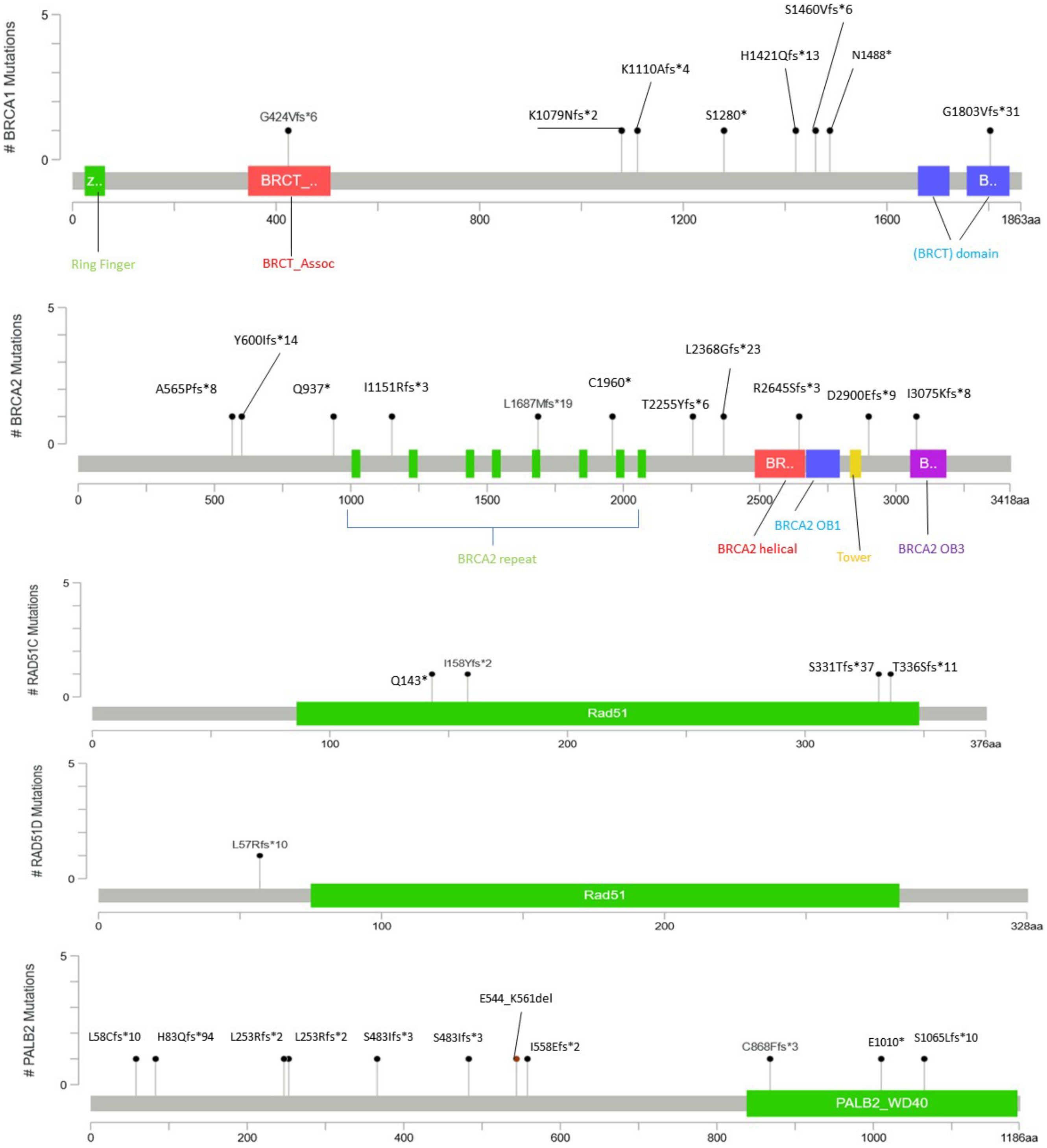

Figure S1. Lolliplot representing BRCA1, BRCA2, PALB2, RAD51C and RAD51D proteins. Novel (likely) pathogenic variants with possible functional impact are presented above the corresponding protein scheme (MutationMapper tool from cBioPortal for Cancer Genomics)
